# Self-Monitoring of Blood Glucose for Patients With Type 2 Diabetes in Primary Care: A Single-Centre, 10-Year Retrospective Analysis

**DOI:** 10.7759/cureus.15597

**Published:** 2021-06-11

**Authors:** Justina Cheh Juan Tai, Liang Zhi Wong, Adrian Richardson

**Affiliations:** 1 General Practice and Primary Care, University College London Medical School, London, GBR; 2 General Practice and Primary Care, NHS Haringey North Central London Clinical Commissioning Group (CCG), London, GBR

**Keywords:** lancets, blood glucose strips, type 2 diabetes mellitus, diabetes, self-monitoring, blood glucose, primary care, general practice, monitoring kits

## Abstract

Background

While type 1 diabetics often require self-monitoring of blood glucose (SMBG), the evidence for tight blood glucose monitoring in non-insulin treated type 2 diabetes mellitus (T2DM) patients is limited. In addition to its lack of cost-effectiveness, unnecessary blood glucose monitoring may also result in anxiety and decreased quality of life. In this retrospective audit, we assessed SMBG prescribing practice at one general practice against guidelines from the National Institute for Health and Care Excellence (NICE).

Methods

A systematic search of T2DM patients diagnosed at a general practice in London, United Kingdom, in the last 10 years was undertaken. A total of 146 patients fulfilled these criteria, of which 100 patients were randomly selected for inclusion in this audit. Medical notes were reviewed and collated for analysis.

Results

Only 85% of patients with T2DM were being managed in accordance with the NICE guidelines on SMBG, while 15% were not. It was more common for patients who did not need monitoring to be inappropriately prescribed SMBG (10%) than it was for patients who needed monitoring to be under-prescribed SMBG (5%). The reasons for prescribing SMBG were often left undocumented.

Conclusion

Adherence to the NICE guidelines is subpar. Recommended solutions include educating healthcare professionals involved in the prescribing of SMBGs, regular reviews of the continued necessity of SMBG, and digital alerts on e-prescribing systems.

## Introduction

Diabetes is recognized by the World Health Organization (WHO) and International Diabetes Federation (IDF) as a major health problem, with about 463 million adults living with diabetes and 4.2 million deaths caused by diabetes in 2019 alone [1]. People with diabetes are at a greater risk of developing cardiovascular, neuropathic, and renal complications, resulting in reduced life expectancy and increased healthcare costs [2].

Self-monitoring of blood glucose (SMBG) was first introduced in the 1960s and has developed significantly since then due to accumulating evidence that regular monitoring of blood glucose is an important part of diabetes management [3]. Knowing how high or low blood glucose levels are would alert the patient to take the necessary corrective steps in managing concerning readings, and thus potentially reduce the risk of long-term sequelae. SMBG also involves patients in their own care, improves patient education and activation, and leads to better glycaemic control outcomes [2,4].

However, while the use of SMBG in patients with type 1 diabetes (T1DM) has been associated with improved health outcomes, the evidence base for the impact of SMBG on patients with type 2 diabetes (T2DM), especially those not on insulin, has been conflicting [5]. Studies conducted have shown little or no correlation between blood glucose monitoring frequency and glycaemic control [6-7]. Moreover, the ‘Efficacy of self-monitoring of blood glucose in patients with newly diagnosed type 2 diabetes’ (ESMON) trial found that although SMBG improved glycated haemoglobin (HbA1c) levels amongst patients with newly diagnosed T2DM, this improvement was not statistically significant [8].

Other studies exploring the effects of SMBG on patients have reported that, while SMBG can empower and motivate patients to be more in control of their health, it can also increase health-related anxiety and depression, and lead to self-blame [9]. This was corroborated by a Cochrane review in 2012, which stated that, in addition to increased anxiety and depression, there was a lack of interest in the results from healthcare professionals, including general practitioners (GP), and a failure to act on the results from both the patients and GPs alike [10]. Additionally, Cameron et al. have shown that SMBG was not cost-effective. They calculated that a 0.25% reduction in HbA1c levels could be achieved when SMBG was performed seven or more times a week, but with an incremental cost of $113,643 per quality-adjusted life-year [11-12].

The National Institute for Health and Care Excellence (NICE) guidance for adults with T2DM was set out in 2015 [13]. It states that SMBG should not be routinely offered to adults with T2DM unless they meet any of the following criteria: (1) the patient is on insulin; (2) there is evidence of hypoglycaemic episodes; (3) the patient is pregnant or is planning to become pregnant; (4) the patient is on oral medication that increases their risk of hypoglycaemic episodes and drives large vehicles or operates heavy machinery. It also recommends considering SMBG when starting patients on treatment with corticosteroids.

This retrospective audit was conducted to determine whether the prescription of SMBG kits at one general practice was in accordance with the NICE guidelines. Data were gathered from online health records to identify if patients currently prescribed blood glucose monitoring kits met the criteria or if any patients who were eligible for SMBG were not offered a kit.

## Materials and methods

This audit was undertaken at a general practice in London, United Kingdom (UK), serving about 4,000 registered patients. Data were collected retrospectively through keyword searches on online patient records on the Egton Medical Information Systems (EMIS) Web. All patients with the clinical code ‘Type 2 Diabetes Mellitus’ first inputted over the period of 10 years (April 12, 2011 - April 12, 2021) were included in this audit. Patients with T1DM, pre-diabetes, gestational diabetes alone, or maturity-onset diabetes of youth (MODY) were excluded. The audit was approved in line with general practice processes.

A total of 146 patients fulfilled the above criteria, of which 100 patients were randomly selected for inclusion in this audit. We only analysed 100 patients due to time and manpower constraints. We carried out simple random sampling to ensure that the 100 patients selected would approximate the results we would have obtained if we had conducted the analysis on all patients. The random sampling was carried out on Microsoft Excel (Microsoft Corporation, Redmond, WA) using the following formula:

=INDEX($A:$A,RANDBETWEEN(1,COUNTA($A:$A)),1)

The 100 included patients were first screened and divided into either: (a) those who were prescribed SMBG strips or (b) those who were not. Individual patient notes and clinic letters were then screened on EMIS Web for SMBG indications based on the NICE guidelines. This was done both manually and through the use of ‘keyword searches’ on the EMIS Web system. To identify patients with a history of hypoglycaemic episodes, the keywords ‘Hypo, Hypoglycaemia and Low glucose’ were used. ‘DVLA, Driver, Drive, Bus, Lorry, Truck, Vehicle and Heavy’ were used to identify patients driving large vehicles or operating heavy machinery. Their medication records were then screened to identify any medication they were currently on that would increase their risk of hypoglycaemic episodes, i.e. sulphonylureas and glinides. ‘Pregnant and Pregnancy’ were the keywords used to screen for patients who were currently pregnant or were planning to get pregnant. The medication records of all patients in the cohort were screened for any use of insulin or courses of corticosteroids.

Results were collated on a spreadsheet with pre-determined headings and explicit criteria to promote standardization between data collectors. If a patient was on SMBG, the type and brand name of their SMBG kit were also noted. The percentage of patients being managed in accordance with the guidelines was calculated. No complex data analysis was performed, so all mean and percentage calculations were made using Microsoft Excel spreadsheet formulations. Ethical approval was not required for this audit, as all aspects of data collection were carried out using an available dataset, and patient information was pooled and not individually disclosed.

## Results

The mean age for this cohort was 64.5 years with a standard deviation of 20.5 years. Patients are further characterised below (Table 1). A wide range of SMBG kits was also found to have been prescribed by this general practice (Table 2). All the known types of SMBG kits prescribed at this practice were compliant with Driver and Vehicle Licensing Agency (DVLA) requirements and International Organization for Standardization (ISO) standards [14].

**Table 1:** Patient characteristics SMBG = Self-monitoring of blood glucose

	Prescribed SMBG	Not prescribed SMBG	Total
N	26	74	100
Mean Age (years)	70.8	62.3	-
Sex (Male)	12	38	50
Sex (Female)	14	36	50

**Table 2:** Types of self-monitoring kits prescribed SMBG = Self-monitoring of blood glucose

Types of SMBG kits prescribed	n
Aviva	2
GlucoRx	14
Contour	2
Fastclix	1
FreeStyle	2
Omni	1
WaveSense	2
Unknown	2
Total	26

**Figure 1:**
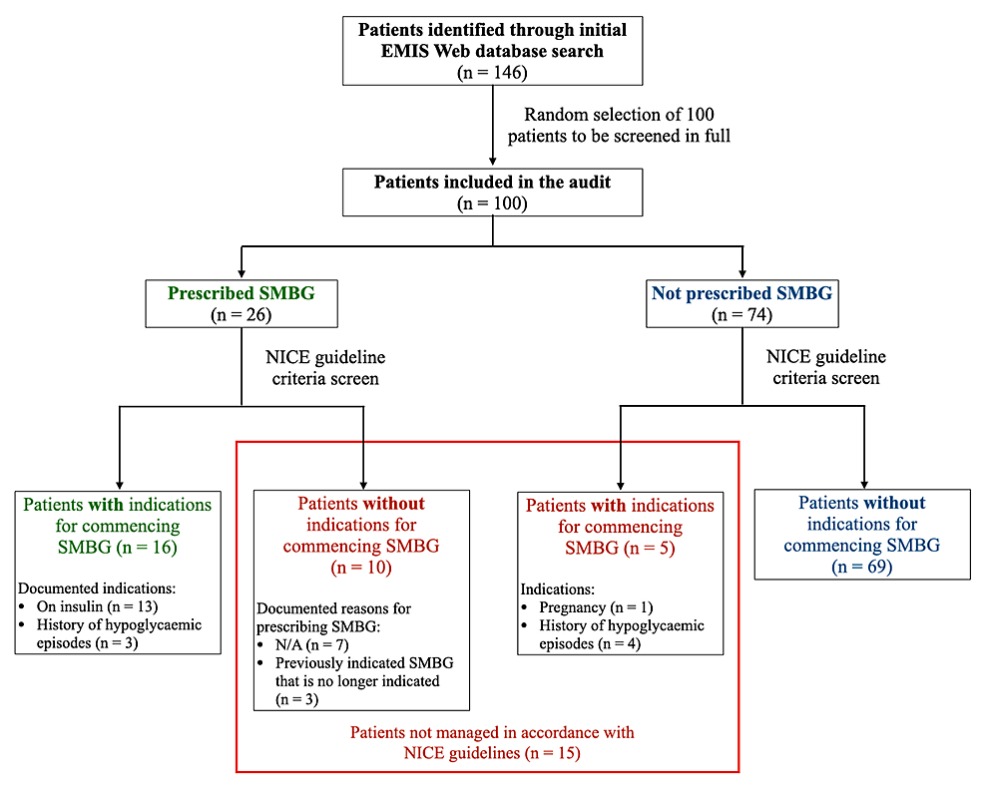
Flowchart describing the audit process and summary of findings EMISWeb = Egton Medical Information Systems Web, SMBG = Self-monitoring of blood glucose, NICE = National Institute for Health and Care Excellence, N/A = Not available/documented

Of the 100 patients, 26 were on SMBG while 74 were not (Figure 1). Of the 26 patients on SMBG, 10 were on SMBG despite having no indication(s) for it. Of these 10 patients, seven patients had no documentation on why they were started on monitoring nor why they were still being monitored. Three patients were on monitoring due to previous recommendations by their respective diabetic clinics for short-term SMBG to monitor the tolerability of new changes in oral medication. All three of these requests had been made over five years ago. Despite this, these patients were still currently being prescribed SMBG despite having tolerated these medication changes well.

**Figure 2:**
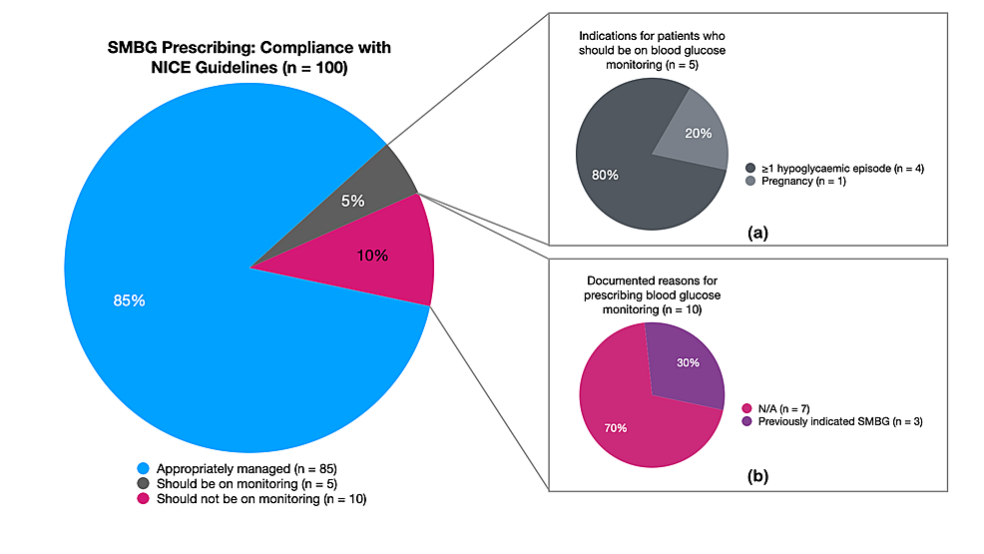
Compliance with NICE guidelines and breakdown of (a) indications for patients who should be on SMBG, and (b) documented reasons for prescribing SMBG in patients who did not need SMBG SMBG = Self-monitoring of blood glucose, NICE = National Institute for Health and Care Excellence, N/A = Not available/documented

Of the 74 patients who were not on SMBG, five patients had at least one indication for starting SMBG. In total, 85% of patients on the register were appropriately managed based on the current NICE guidelines while 15% were not (Figure 2).

## Discussion

Main findings

This retrospective audit found that only 85% of patients with T2DM were being managed in accordance with the NICE guidelines on SMBG while 15% were not. A total of 10% of patients did not require SMBG but were being regularly prescribed SMBG while 5% of patients had indications for SMBG but were not prescribed SMBG.

The literature on SMBG demonstrates the fine balance that has to be struck when prescribing SMBG to non-insulin treated T2DM patients. For a small proportion of patients, SMBG may be beneficial in warning patients of impending hypoglycaemic episodes or providing reassurance before the use of heavy vehicles and machinery. For pregnant patients, SMBG screens for less-than-ideal glucose levels that may need to be optimised for the duration of pregnancy.

However, for the majority of patients who do not fall into the aforementioned categories, SMBG is neither recommended nor justified. While there have been older reports of improved HbA1c levels following SMBG among non-insulin treated T2DM patients [15-16], more recent systematic reviews have since found either non-statistically significant changes in glycaemic control [17] or very limited demonstrable clinical benefit [18-20]. Patients are not trained on how to use the information from their SMBG readings to make adjustments in their oral therapy, and thus, these SMBG readings do not actually have any impact on management in the short term. SMBG as a monitoring tool itself is far from perfect, as evidenced by large fluctuations in readings throughout the day depending on the timing of the measurement in relation to the level of activity, food intake, and timing of medication [21]. Unless SMBG is performed extensively throughout the day, the readings obtained are arbitrary at best and are unlikely to be meaningful; one-off readings without vital context can lead to unnecessary anxiety, or conversely, false reassurance [5,9]. On the other hand, rigid and rigorous use of SMBG is unhelpful and has been shown to reduce the quality of life [22].

To the best of the authors’ knowledge, this is the first study to evaluate SMBG management in primary care against the current NICE guidelines. An unexpected finding from this audit was the number of patients who were on long-term SMBG without any indications. Possible reasons for this issue are as follows: (1) poor awareness of national and local SMBG guidelines; (2) annual medication reviews that focus on pharmacological interventions rather than on supplementary monitoring devices; (3) patient dependence on SMBG due to long-standing habits; (4) the prioritisation of other, arguably more dire, aspects of patient care due to existing high pressures on primary care services. It must be recognised that population-based guidelines and measures can have vast amounts of variability and applicability on an individual level, so there will be exceptions that should be managed at the discretion of the prescribing healthcare professional. However, when this is the case, the reasoning should be justified and clearly documented in the notes. Indeed, it was observed that once SMBG had been commenced for the first time, blood glucose testing strips often turned into automatic ‘repeat prescriptions’ and were rarely reviewed again. There were a small number of patients who were on SMBG due to recommendations from diabetic clinics in secondary care, all of which were due to a change in their drug regimen. However, in these cases, the duration for which patients needed to be performing SMBG was never specified; this resulted in patients who had successfully tolerated regimen changes and were now on established treatments to still be on SMBG despite the fact that it was no longer necessary. This highlighted another aspect of the guidelines that was often not complied with - the need to “carry out a structured assessment [of the continued benefit of self-monitoring] at least annually” [13].

The unnecessary prescribing of SMBG has been a deep-seated issue for years; prior to the introduction of the NICE guidelines, Robson et al. conducted a multi-centre study with three London-based clinical commissioning groups (CCG) in the years 2010-2013, investigating SMBG prescribing habits in primary care [23]. They found that while the trend of inappropriate SMBG use was already decreasing over time without any intervention, they were able to significantly accelerate the reduction of inappropriate SMBG use with the introduction of a multi-faceted programme involving local guideline development, education, and IT support. They estimated a potential £21.8 million per annum savings in diabetes prescribing costs and the prevention of unnecessary SMBG in 340,000 people if their programme had been replicated nationally. Although awareness of this issue has greatly increased since then, largely due to the development and dissemination of national guidance, this audit shows that clinical practice still lags behind, even six years after the guidance was first issued.

It was also interesting to note, as a secondary finding, the lack of standardisation in types of blood glucose kits prescribed. Freckmann et al. highlighted some variation in the quality and accuracy of various monitoring systems [24], and while all SMBG kits prescribed in this general practice were compliant with DVLA requirements and ISO standards, it is clear that not all SMBG kits are equal. In particular, the price variation between testing strips for the kits prescribed were vast and ranged from £5.45 (GlucoRx Q, GlucoRx Ltd, Guildford, United Kingdom) to £16.40 (FreeStyle, Abbott Laboratories Ltd, Illinois, United States) per 50 strips [25].

The reasons for the lack of compliance with the guidelines and variation in practice must be ascertained, as these issues collectively come at a substantial cost, both financially and to the patient’s quality of life. Recommended solutions include the education of healthcare professionals involved in the prescribing of SMBGs, regular reviews of the continued necessity of SMBG, and digital alerts on e-prescribing systems to remind healthcare professionals to be conscious of SMBG guidelines when dealing with non-insulin-treated T2DM patients. A re-audit will be highly informative once ample time has passed to allow the effect of these changes to be embedded into practice.

Limitations

While this audit included a substantial sample of patients, the data lack external validity, as all the patients were recruited from a single centre. Information was also gathered through a review of patient records, rather than through surveys or interaction with patients, so there were several factors that were not accounted for: (1) whether patients were using the SMBG kits that they were prescribed; (2) whether patients who were not prescribed SMBG had obtained their own kits and lancets over-the-counter; (3) whether indications for SMBG existed, but were not documented, by the prescribing healthcare professional. The generalisability of this study is also limited due to the inevitable variation in patient demographics, socioeconomic status, and health literacy between CCGs across the country. As this was the first audit to investigate adherence of primary care professionals to the NICE guidelines on SMBG, there was no ‘ideal standard’ to which the results of the audit could be compared to or corroborated with.

## Conclusions

The role of SMBG in the management of non-insulin-treated T2DM is clearly delineated in the NICE guidelines, but this audit revealed that adherence to these guidelines was less than 100%. Other CCGs are urged to consider performing similar audits on local SMBG prescribing practice, as it may well be that these findings are replicated in other parts of the UK, pointing to a far wider-reaching issue than demonstrated by this single-centre audit alone. In terms of future research, it would also be worth further exploring the effect of reduced SMBG on outcomes such as glycaemic control, symptomatic episodes of hypoglycaemia, diabetes-related morbidity and mortality, and quality of life. In the spirit of patient-centred care, qualitative research exploring more open-ended, patient-driven outcomes should also be carried out to ascertain patient views and acceptability of (or, rather, lack of) SMBG. Newer alternatives, such as continuous blood glucose monitoring (CGM) and non-invasive glucose monitoring, may be explored as potential options for patients who do not meet the criteria for SMBG but who may benefit from less intrusive monitoring.
